# The state of the art in student engagement

**DOI:** 10.3389/fpsyg.2015.00355

**Published:** 2015-03-27

**Authors:** Carl Senior, Chris Howard

**Affiliations:** ^1^Psychology, School of Life and Health Sciences, Aston UniversityBirmingham, UK; ^2^Department of Psychology, University of DerbyDerby, UK

**Keywords:** student engagement, student learning outcomes (SLOs), relational learning, social behavior, learning technology

There has been a considerable shift in the higher education literature from a focus on the characteristics and traits of individual students to the role of the learning environment. More than ever, the context in which learning takes place has come to the fore and the role of lecturers in helping to facilitate learning and to engage students across a wide diversity of learning contexts is of upmost importance. To this end, learning is relational, which involves the development and quality of relationships between students and lecturers but also the relationships between students themselves (see Gergen, 2011). The empirical evidence is encouraging as it demonstrates that students who learn collaboratively achieve higher grades than students working independently. The challenge for programme managers is to move beyond the prescriptive view that learning only takes place at an individual level within the lecture theater, seminar or tutorial room and explore the wider sets of relationships and communities in which students are situated within (Singh, 2003; Garrison and Vaughan, 2008). Students come to higher education institutions already engaged in a wider set of relationships (e.g., family, employment, and organizations) and will develop new ones through their studies which need to be understood in order to engage students with innovative program design and delivery. This research topic was born out of this need and collects together a range of perspectives that converge on one salient question i.e., by what means can we further engage students in their studies?

This research topic is both innovative and ambitious and highlights as well as consolidates the current understanding of the role that student based engagement behaviors may serve in effective pedagogy. Of the nine papers submitted six were platform articles that highlighted an existing agenda or set a further direction for new lines of work. The very fact that so many opinion articles were submitted to this topic perhaps highlight the need for more empirical work in this area.

In a highly original article, Irving (2015) describes her work on the use of dance to engage students with the development of statistical literacy. Research methods and statistics are traditionally viewed as difficult topics by undergraduate psychology students and many fail to engage due to the (miss) perceived difficulty that they may encounter (Onwuegbuzie and Wilson, 2010). However, by communicating statistical concepts via the medium of dance Irving argues that students can be more readily engaged within this topic. A similar process is advocated with the work of McGivern and Coxon (2015) as well Rich et al. (2014) who respectively describe the possible role that student polling software and student focused assessments have in driving engagement and retention. While Orosz et al. (2015) found that teacher enthusiam drove a reduction of cheating behaviors in subsequent assessments. These articles each highlight the need for student-focused activities in the classroom as facilitators of engagement.

However, such student-focused activities need not be purely designed around the manner in which students engage with their respective programme. Indeed, Senior et al. (2014b) argue that the unique experience of maintaining gainful employment at the same time as studying full time should be considered as a central part of programme design. Not only will this ensure that subsequent programmes are flexible enough to support the real world needs of the incoming student cohort but they will also be designed to facilitate effective learning with students who often have to balance the needs of full time study with work and sometimes even family commitments (see also Senior and Cubbidge, 2010). Such student-facing commitments are significant and as Gill et al. (2015) describe sometimes they are simply essential to consider in the delivery of programmes that maximize student retention. While, Senior et al. (2014a) make an interesting observation and bring in organizational theory to highlight the importance of differences in the style of academic leadership and how a more open and relationship focused style of leadership may have a significant benefit in driving student retention.

Taken together these opinion papers describe the importance of various factors that when considered may increase student retention and maximize engagement—these are the gold standards of effective programme design. The work by Hammar-Chiriac (2014), Orosz and colleagues (2015) as well as Senior and Howard (2014) examine the possible social mechanisms that are in play in driving such engagement. Hammer-Chiriac first examines the social processes that are experienced during the act of engaging with group work. In a study that spanned across two institutes involving several programmes and analysis was carried out to examine the student experiences of group work. Three key factors were uncovered to play a significant role in student engagement these being the organization of the group, its effective role in facilitating learning and also its function in the facilitating and an affiliation to a particular discipline based group. Here the students use group work to develop and enforce their emerging professional identity. This goes beyond subscribing to a professional identity merely by enrolling on a particular programme here the act of working together on a group project actively drives the formation of such a professional identity.

The social psychological mechanism behind such a process was examined further in the work by Senior and Howard (2014). In a series of focus group carried out with students who enrolled on Psychology programmes it was revealed that students used their immediate friendship groups not only to reinforce their professional identity but to also reinforce their own understanding of the topics that had been discussed in their lectures. However, perhaps more interestingly was the fact that those student who used their friendship groups to reinforce their understanding of lecture topics were also unaware they were doing so. The authors of this paper highlight the fact that the students were using their engagement within a so called community of learners as an effective learning mechanism but also coined the phrase the “implicit community of learners” to describe the manner in which the students were engaging with their social groups to reinforce their professional identity as well as develop a stronger understanding of the disciplinary concepts.

All of the work described above is a snap shot of the current state of the art in student engagement highlights the various means by which future work can make an impact. We would like to express our sincere thanks to all of the reviewers for the papers submitted to this Research Topic and to Professors Jesus De La Fuentes and Jason Osborne who agreed to act as action editors for the papers that we submitted—without them the work you are currently reading will not have happened.

## Conflict of interest statement

The authors declare that the research was conducted in the absence of any commercial or financial relationships that could be construed as a potential conflict of interest.
